# Metallic conduction through van der Waals interfaces in ultrathin $\hbox{Bi}_2\hbox{Te}_3$ films

**DOI:** 10.1038/s41598-021-85078-9

**Published:** 2021-03-11

**Authors:** Shinichiro Hatta, Ko Obayashi, Hiroshi Okuyama, Tetsuya Aruga

**Affiliations:** Department of Chemistry, Graduate School of Science, Kyoto University, Kyoto, 606-8502 Japan

**Keywords:** Surfaces, interfaces and thin films, Two-dimensional materials

## Abstract

While the van der Waals (vdW) interface in layered materials hinders the transport of charge carriers in the vertical direction, it serves a good horizontal conduction path. We have investigated electrical conduction of few quintuple-layer (QL) $\hbox {Bi}_2\hbox {Te}_3$ films by in situ four-point probe conductivity measurement. The impact of the vdW (Te–Te) interface appeared as a large conductivity increase with increasing thickness from 1 to 2 QL. Angle-resolved photoelectron spectroscopy and first-principles calculations reveal the confinement of bulk-like conduction band (CB) state into the vdW interface. Our analysis based on the Boltzmann equation showed that the conduction of the CB has a long mean free path compared to the surface-state conduction. This is mainly attributed to the spatial separation of the CB electrons and the donor defects located at the Bi sites.

## Introduction

Layered materials consisting of two-dimensional (2D) atomic frameworks have attracted much attention for their potential applications in future devices based on hetero- and nano-structures^1–3^. Because each layer has no dangling bonds, interlayer attractive force is attributed to the van der Waals (vdW) interaction. Although electronic coupling at the vdW interface is weak, it can play a subtle but significant role in the band formation and physical properties of few-layer films. A well-known example is the direct–indirect gap transition between monolayer and multilayer $\hbox {MoS}_2$ films^4,5^.

**Figure 1:**
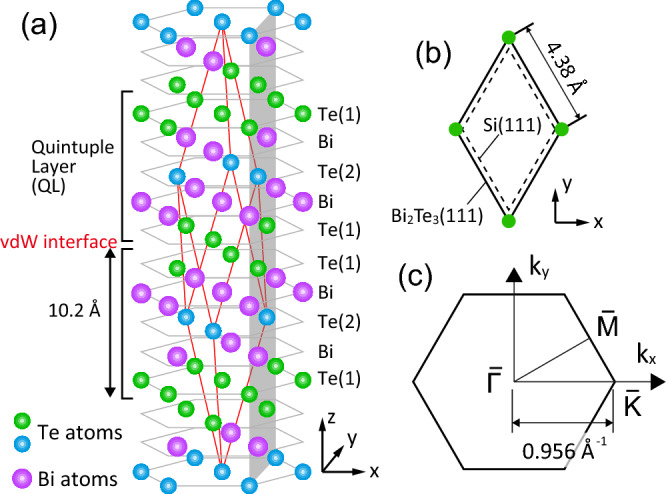
(**a**) Bulk crystal structure of $\hbox {Bi}_2\hbox {Te}_3$. The (red) parallelepiped represents a rhombohedral unit cell. (**b**) $\hbox {Bi}_2\hbox {Te}_3$ and Si (111) unit cells. (**c**) A 2D Brillouin zone of $\hbox {Bi}_2\hbox {Te}_3$(111).

$\hbox {Bi}_2\hbox {Te}_3$ is a member of V-VI layered compounds ($\hbox {Bi}_2\hbox {Se}_3$, $\hbox {Sb}_2\hbox {Te}_3$, etc). The bulk crystal structure is composed of triangular-lattice Bi and Te layers stacked along [111] of the rhombohedral lattice (Fig. 1a). A set of two Bi and three Te layers, a quintuple layer (QL), is a building block of the layered structure. The stacking of the atomic layers is an ABC type (see also Fig. 5b), and thus, a rhombohedral unit cell contains three QLs. The outer Te layer (Te(1) in Fig. 1a) of a QL is adjacent to the equivalent one of the neighboring QL. The Te–Te interatomic distance^6^ between the adjacent layers (3.65 Å) is much longer than the Te–Te covalent bond length^7^ of 2.7 Å, which indicates vdW-type interaction at a QL–QL interface.

Electronic properties of $\hbox {Bi}_2\hbox {Te}_3$ have been extensively studied since it is an important material exhibiting a high thermoelectric power factor around room temperature^8^, and, recently, it was found that the (111) surface possesses topological surface states (TSS) characterized by novel spin texture and Dirac-cone-like dispersion^9–11^. Although $\hbox {Bi}_2\hbox {Te}_3$ is a narrow-gap semiconductor, metallic conduction is often observed for melt-grown crystals and deposited thin films, which is often ascribed to native antisite defects^12–17^. Synthesis of ultrathin films of $\hbox {Bi}_2\hbox {Te}_3$ is an effective way to reduce the bulk conduction and hence to extract the contribution of TSS^18–21^. On the other hand, with decreasing thickness, the decoupling of TSS localized on opposite surfaces is altered below a critical thickness, resulting in band gap opening^18,20^. Previous studies showed that the 4-QL film was found to be thick enough to host bulk-like gapless TSS. Below this thickness, critical difference in band structure near the band gap was indicated between 1- and 2-QL films. On the other hand, the film thinning of $\hbox {Bi}_2\hbox {Te}_3$ was reported to enhance thermoelectric power^22,23^. However there is little experimental evidence about transport properties in films with thickness below 5 QL.

In this study, we investigated electronic and transport properties of a few-QL $\hbox {Bi}_2\hbox {Te}_3$ films by combining angle-resolved photoelectron spectroscopy (ARPES), four-point-probe (4PP) conductivity measurement and first-principles band calculation. The ultrathin films with thickness of 1–5 QL exhibited metallic electrical conduction. A large increase in sheet conductivity was observed from 1 to 2 QL due to the formation of a bulk-like conduction band (CB), whose electron density is highly concentrated at the vdW interface. The localization of the CB state prevents conducting electrons from being scattered by the Bi antisite defect.

## Results and discussion

**Figure 2:**
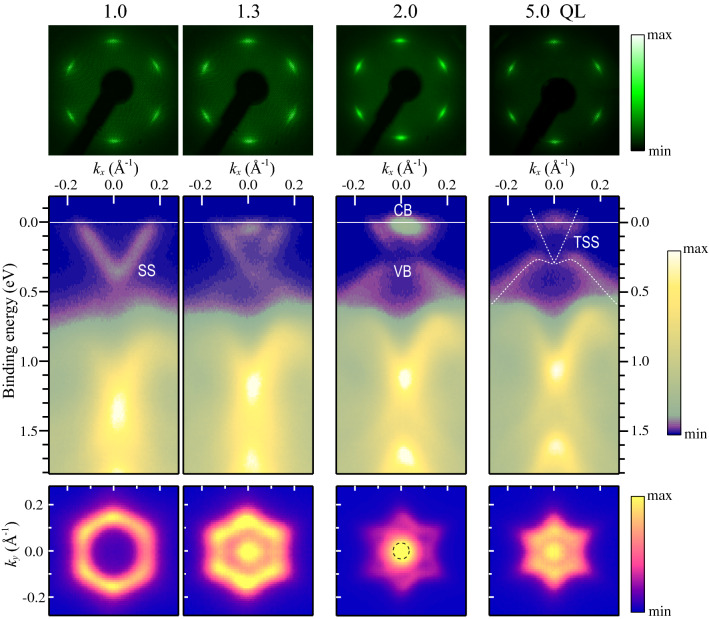
(Top panels) LEED patterns at 70 eV, (middle)ARPES band maps and (bottom) Fermi surface maps of the $\hbox {Bi}_2\hbox {Te}_3$ films at 1.0, 1.3, 2.0 and 5.0 QL. The Fermi surface maps were obtained by mirroring the data at $k_y=0$. The dashed circle on the bottom panel at 2 QL represents the Fermi surface of the bulk-like CB. The dotted curves on the middle panel at 5 QL represent the dispersions of the TSS and VB bands.

The top panels of Fig. 2 show low-energy electron diffraction (LEED) patterns observed for the $\hbox {Bi}_2\hbox {Te}_3$ films of 1, 1.3, 2 and 5 QL. The in-plane lattice constant is evaluated to be 4.34±0.04 Å, which agrees well with that of the bulk $\hbox {Bi}_2\hbox {Te}_3$(111) plane (4.38 Å, Fig. 1b). The six-fold LEED pattern indicates double-domain $\hbox {Bi}_2\hbox {Te}_3$ films with opposite stacking sequence. Each $\hbox {Bi}_2\hbox {Te}_3$ spot has an arc shape, but is sharp along the radial direction, which shows that large domains of the ultrathin films are epitaxially grown on Si(111) with distribution of domains rotated by $\le 4.9^{\circ }$ about the [111] axis.

The middle and lower panels of Fig. 2 show ARPES band and Fermi-surface maps around $\bar{\Gamma }$. The $k_x$ axis is along $\bar{\Gamma }$–$\bar{K}$ (Fig. 1c). The 1-QL film has a metallic band with V-shaped dispersion. The band labeled SS has a hexagonal Fermi surface and the minimum energy of 0.34 eV at $\bar{\Gamma }$. The SS band energy was found to be the same between the films on n-type Si(111) (Fig. 2) and on p-type Si(111) (Fig. 4), and thus, it is independent of the substrate Fermi level ($E_{{\rm F}}$) position. With increasing thickness, two additional features grow at $E_{{\rm F}}$ and 0.3–0.5 eV. The upper and lower bands are attributed to bulk-like CB and valence band (VB), respectively. The CB yields a small Fermi circle whose radius is evaluated to be 0.034 Å$^{-1}$. Although the evolution of the SS band dispersion is not clear in the band maps, the Fermi surface changes into a warped hexagon at 1–2 QL. The Fermi surface at 2 QL is already very similar to that of TSS on the bulk surface. The clear difference in Fermi-surface shape between 1 and 2 QL indicates that the $\hbox {Bi}_2\hbox {Te}_3$ film grows QL by QL. For further deposition up to 5 QL, no additional band feature is seen, but the position of $E_{{\rm F}}$ is slightly shifted downward toward the band gap between VB and CB. The shift induces the contraction of the Fermi surfaces of SS and CB by about 20%. Since the VB maximum is located at $0.27\pm 0.02$ eV for the 5-QL film, the Dirac point of TSS is estimated to be at 0.3 eV. The behavior observed at 1–5 QL is consistent with previous reports^18,20^, and demonstrates that the few-QL films have one or more metallic states.

**Figure 3:**
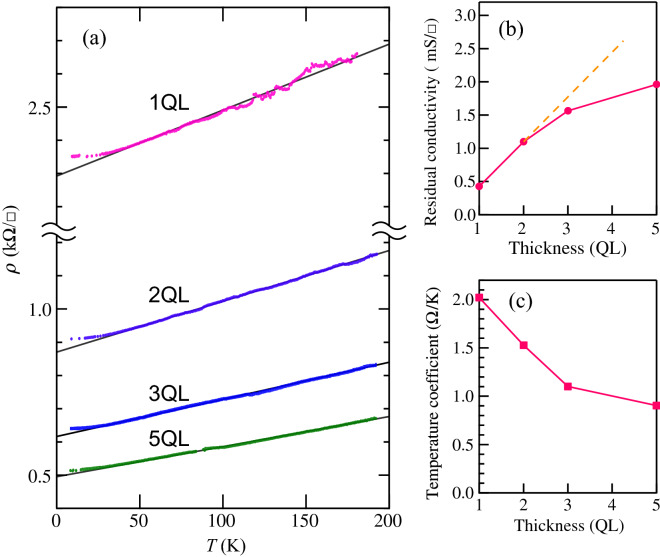
(**a**) Temperature dependence of $\rho$ of the 1-, 2-, 3-, and 5-QL $\hbox {Bi}_2\hbox {Te}_3$ films measured during heating from 9 K. The fitting lines are depicted by the solid lines. The symbol /$\square$ in the vertical axis label is used to differentiate sheet resistivity $\rho$ from resistance. Thickness dependence of (**b**) residual sheet conductivity and (**c**) linear temperature coefficients of $\rho$(*T*). The dashed line in (b) represents the expectation as conductivity increases with the number of the vdW interfaces.

Figure 3a shows the temperature dependence of sheet resistivity of the $\hbox {Bi}_2\hbox {Te}_3$ films of thicknesses of 1, 2, 3 and 5 QL. We used low-doped Si(111) substrates ($\rho _{3D}>1000\ \Omega \,\hbox {cm}$, n-type) in the conductivity measurements. While the conductivity in the Si bulk and surface space-charge layer rapidly drops in the low temperature (freeze-out) range^24,25^, no sign of it is seen in all the $\rho$–*T* curves, which indicates the contribution of electron conduction in the substrate is negligible. Hence, the $\rho$–*T* curves evidence the metallic nature of the few-QL films. On the other hand, electron transport in ultrathin films is affected by substrate atomic steps and surface contaminations. In our study the 1-QL film must be most sensitive to scattering by such extrinsic factors. However the variation in resistivity of 1-QL films was within $\sim$10%, which is sufficiently small compared to the thickness-dependent decrease in resistivity. Linear functions fitted to the data at $T\ge 50$ K are displayed by the thin lines. The linearity represents the dominant role of electron–phonon (*e*–ph) scattering in the high temperature regime. With decreasing temperature below 50 K, the $\rho$–*T* curves begin to deviate from the linear lines, and become nearly flat below $\sim$20 K. From the $\rho$–*T* curves, we evaluated residual sheet conductivity $\sigma _0$, the inverse of $\rho$ measured at 9 K, and temperature coefficient $d\rho /dT$ in the high temperature regime. They are displayed as a function of film thickness in Figs. 3b and 3c. According to the ARPES result, the $\sigma _0$ value of the 1-QL film is directly attributed to the SS band; $\sigma _{0}^{{\rm SS}}=0.43$ mS. The conductivity increase at 2 QL can be related to the formation of CB or the change of the SS band. Actually, the hexagonal Fermi surface at 1 QL and the warped one at 2 QL have a tiny difference of at most 3% in length of Fermi contours, to which carrier density in 2D is proportional. We therefore attribute the increase in $\sigma _{0}$ between 1 and 2 QL to the CB Fermi surface; $\sigma _{0}^{{\rm CB}}=0.67\,\hbox {mS}$.

We analyze the transport properties of the SS and CB bands by using the Boltzmann equation of 2D electrical conductivity:

1 $$\begin{aligned} \sigma = \frac{e^2\tau }{2\pi ^2 \hbar }\int \frac{v_{kx}^2}{|{\varvec{v}_{k}}|} dk, \end{aligned}$$

where ${\varvec{v}_{k}}$ is a group velocity, the integral is done on Fermi contours, and $\tau$ is a carrier relaxation time. The $k_{x}$ direction here is defined along $\bar{\Gamma }\bar{M}$, parallel to the array of 4PP. The scattering rate is the sum of the rates of defect scattering and phonon scattering: $\tau ^{-1} = \tau _{0}^{-1}+\tau _{ph}^{-1}$. The first term determines $\sigma _0$, and the second term describes the constant $d\rho /dT$ slope as $\tau ^{-1}_{ph}=2\pi \lambda _{tr}k_{{\rm B}}T/\hbar$, where $k_{{\rm B}}$ is the Boltzmann constant and $\lambda _{tr}$ a transport *e*-ph coupling constant. The group velocity was deduced from the band dispersions at $E_{{\rm F}}$. For the SS band, we obtained $v_k=0.39\times 10^8\,\hbox {cm/s}$ along $\bar{\Gamma }\bar{M}$ and $0.48\times 10^8\,\hbox {cm/s}$ along $\bar{\Gamma }\bar{K}$ from ARPES. Because the dispersion of the CB is not clear in the ARPES data, we used a theoretical value of $0.41\times 10^8\,\hbox {cm/s}$ for the 2D free-electron like Fermi surface.

We obtained $\tau =17$ fs from $\sigma _{0}^{{\rm SS}}=0.43$ mS, and $\lambda _{tr}=0.06$ from $d\rho /dT=2.02 \Omega /\hbox {K}$ at 1 QL. The magnitude of $\lambda _{tr}$ is in a range of the experimental and theoretical values (0–0.1) for the surface of bulk $\hbox {Bi}_2\hbox {Te}_3$^26–28^. A mean free path *l* was evaluated to be 7.4 nm by multiplying $\tau$ and averaged $v_k$. On the other hand, we obtained $\tau =120$ fs and $l=49$ nm for the CB at 2 QL, which are much longer than those for SS. The evaluation of $\lambda _{tr}$ from a band-averaged $\tau$ gives $\lambda _{tr}=0.05$, which is close to that in the 1 QL film. It is very likely that the $\lambda _{tr}$ is nearly independent of thickness, and the decrease of $d\rho /dT$ with thickness is due to the increase in $\tau _{0}$ and the additional contribution of the CB to the integral in eq. (1). This qualitatively explains why the curves of $d\rho /dT$ in Fig. 3c and $\sigma _{0}$ in 3b are similar but upside down.

**Figure 4:**
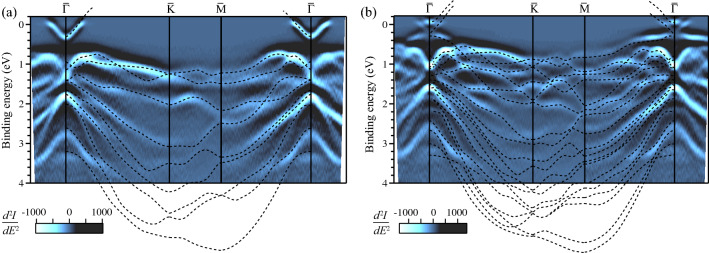
Measured band structure of the (**a**) 1-QL and (**b**) 2-QL $\hbox {Bi}_2\hbox {Te}_3$ films along $\bar{\Gamma }$–$\bar{K}$–$\bar{M}$–$\bar{\Gamma }$. These are second-derivative ARPES intensity maps. Lower negative second derivatives ($d^2I/dE^2$) are colored brighter to highlight peaks in energy distribution curves. The dashed curves are calculated band structure of the free-standing 1-QL and 2-QL $\hbox {Bi}_2\hbox {Te}_3$ films.

In order to gain insight into the significant difference in transport property between the SS and CB bands, we explore the nature of the two states by first-principles calculations. Firstly, we compare the computed band structure of free-standing $\hbox {Bi}_2\hbox {Te}_3$ films with the measured one. Figures 4a and 4b show the ARPES band maps along $\bar{\Gamma }$–$\bar{K}$–$\bar{M}$–$\bar{\Gamma }$ measured on the 1-QL and the 2-QL films, respectively. The calculated electronic structure, which is expanded in energy by a factor of 1.09 to compensate an underestimate of band energies in DFT calculations, is displayed by the dashed curves. For the 1-QL film, the number of calculated valence bands is 9, which corresponds to the sum of six 6p electrons from Bi atoms and twelve 5p electrons from Te atoms. The details of theoretical band dispersions show an excellent agreement with the experimental ones for both films over the whole 2D Brillouin zone.

**Figure 5:**
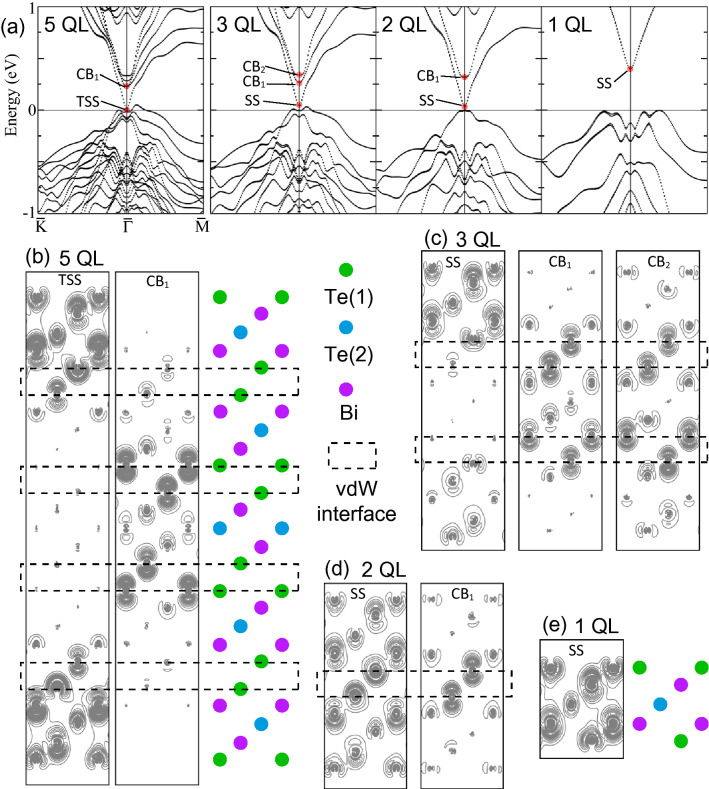
(**a**) Calculated band structure of the 5-, 3-, 2- and 1-QL films. For the states (TSS (SS) and CB states) marked in (**a**), electron density contour maps are drawn in (**b**–**e**) on the *y*–*z* plane (the gray parallelogram in Fig. 1a). The dotted rectangles indicate the vdW interfaces.

Figure 5a shows the calculated band structure in the vicinity of $E_{{\rm F}}$ for the bulk-like 5-QL and thinner 3-, 2- and 1-QL films. On the 5-QL film, the TSS band contacts with the VB at $\bar{\Gamma }$ and $E=0$ eV. At higher energies, there are four CBs. While the TSS band and the CB have similar dispersion relations, their electron density distribution is different as shown in Fig. 5b. The TSS is confined in the outmost QLs and has considerably small electron density in the inner three QLs, which is the evidence of the decoupling of TSS on the both outmost QLs. On the other hand, a CB state (CB$_1$) exhibits a high concentration of electron density on the Te(1) atoms faced with two middle vdW interfaces. The other CB states also show electron density peaks at vdW interfaces with different distributions. Moreover, it should be noted that the CB states is mainly derived from the Te(1) orbitals in contrast to the SS with comparable contributions from Te and Bi orbitals. For the thinner films, a band gap is seen at $E=0$ eV, indicating a transition from nontrivial TSS to SS. As to CBs and VBs, their numbers are decreased as $n-1$, where *n* is the number of QL. The localization of the CB states into vdW interfaces is preserved as shown in Fig. 5c, d. For the 1-QL film, only an unoccupied band is seen. Its electron density distribution (Fig. 5e) has the common nature with the SS (TSS) rather than the CB state.

We now turn back to the transport properties of the SS band and the CB. Relaxation time was evaluated from the conductivity measured at 9 K. In the low-temperature regime, phonons freeze out. On the other hand, donor sites are still ionized, which is confirmed by the metallic behavior of $\rho$ in Fig. 3a. Metallic conduction with n-type carriers was observed for Te-rich $\hbox {Bi}_2\hbox {Te}_3$ samples^12,14^. DFT calculations showed the lowest formation energy of the Bi antisite defect^15,16^, at which a Bi atom is substituted by Te. The calculation showed a defect-induced resonance lying just above the CB minimum^15^. This is consistent with the fact that metallic conduction was observed at low temperatures. The comparison of the electron density maps of the SS and CB states reveals that the conduction through the SS band is affected more strongly by the Bi antisite defects than that through the CB. We therefore attribute the shorter relaxation time for the SS to the scattering by the Bi antisite defects. Because the Bi antisite defect is a single electron donor, we obtained for the 2-QL film a defect density, $n_{d}=1.83\times 10^{12} /\hbox {cm}^{2}$ from the area of the observed CB Fermi surface. The average distance of the antisite defects is estimated to be $\sqrt{1/n_{d}}\sim 7$ nm, which is comparable with the mean free path for the SS conduction (7.4 nm).

The band calculations of the few-QL films also provide a simple description of the conductivity above 2 QL. The increase in number of the CB with thickness causes the increase in carrier density. Assuming the common dispersion of the CB states, the conductivity of the *n*-QL film is expressed as $\sigma (n)=\sigma_{{\rm SS}}+(n-1)\sigma_{{\rm CB}}$, which is shown by the dashed line in Fig. 3b. This expression is qualitatively consistent with the total conductance in a parallel circuit consisting of the vdW interface and the surface. The estimated $\sigma (3)$ (1.8 mS) roughly agrees with the measured $\sigma _0=1.56\,\hbox {mS}$. Actually, ARPES showed that the CB minimum is shifted upward from 0.1 eV below $E_{{\rm F}}$ with increasing thickness. In addition, the calculated CB states for $\gtrsim$ 3 QL have energy spread of 0.05–0.1 eV at $\bar{\Gamma }$. Therefore the conductivity increase due to the CB must be limited above 3 QL. On the other hand, the transition from SS to TSS at 5 QL is expected to induce a large increase in conductivity because carrier backscattering for the TSS is suppressed by spin-momentum locking. In the previous study, a very high mobility for TSS ($4600\,\hbox {cm}^2/\hbox {Vs}$ at 14 K) was shown for $\hbox {Bi}_2\hbox {Te}_3$ single-crystalline ultrathin films^21^. However the conductivity increase observed from 3-QL to 5-QL films is only 0.5 mS, which is smaller than those by the CB at 1–2 QL and 2–3 QL. This result is attributed to the coexistence of inversely-stacked domains in our $\hbox {Bi}_2\hbox {Te}_3$ films. The spin polarization of TSS is reversed between surfaces of inversely-stacked domains, and thus, the domain boundary strongly hinders the transport of the TSS carriers.

Finally we describe the anisotropy of the carrier transport of $\hbox {Bi}_2\hbox {Te}_3$^29,30^. For the 2-QL film, a mobility of $2300\,\hbox {cm}^2/\hbox {Vs}$ was obtained from a CB carrier density $n_{d}$ and $\sigma _{0}^{{\rm CB}}$. This value is considerably high compared with $300\,\hbox {cm}^2/\hbox {Vs}$ reported for 500-nm-thick films with a similar n-type carrier concentration^14^. While the mobility measured for the thicker films contains both in-plane and out-of-plane conduction, our measurement of the 2-QL film evaluates only the in-plane conduction. Therefore the high mobility for the 2-QL film is related to the strong anisotropy of the CB carrier transport as reported for bulk single crystals^29^. Nevertheless, taking into consideration the critical role of domain boundary scattering in electrical conduction in ultrathin films, the high mobility is surprising. We note that the mean free path (49 nm) for the CB is longer than the mean domain size (6–8 nm) evaluated from the LEED spot width in the radial direction (Fig. 2). This suggests a low barrier for CB carrier transport across the domain boundaries.

## Conclusions

In summary, we have shown the critical role of the vdW interface in electronic structure and electrical conduction in the few-QL $\hbox {Bi}_2\hbox {Te}_3$ films grown by MBE. We observed the large increase in electrical conductivity with the formation of the bulk-like CB associated with the vdW interface. Although TSS is not preserved in the $\hbox {Bi}_2\hbox {Te}_3$ films with thickness below 4 QL, the CB state exhibits high sheet conductivity and mobility due to the separation of conducting electrons from the donor defect site. The localized nature of the CB state is expected to be effective for reducing the influence of surface contamination by adsorbates or a capping layer. This property is useful for fabricating robust atomically-thin metallic films or wires. Finally, it should be noted that the vdW interface state for the 2-QL film is confined within only two-atomic-layer thickness as shown in Fig. 5d. The interface electrons will provide a new platform for studying quantum properties of 2D electrons. For example, the evolution of 2D magnetic order mediated by the 2D metallic state can be addressed by the intercalation of magnetic atoms, such as Cr^31^, into the vdW interface.

## Methods

Few-QL $\hbox {Bi}_2\hbox {Te}_3$ films were grown by molecular-beam epitaxy (MBE) with a polycrystalline $\hbox {Bi}_2\hbox {Te}_3$ source in an alumina crucible heated at 700 K. As a substrate, we used the Bi/Si(111) $\beta$–$\sqrt{3}\times \sqrt{3}$ surface where the dangling bonds on Si(111) are fully terminated by Bi adatoms^32^. A typical growth rate of 0.2 QL/min was obtained on the substrates at room temperature while keeping back-ground pressure below $5\times 10^{-8}$ Pa. Deposited films were annealed at 370 K for 1 min. The moderate postannealing reduced the background intensity of LEED, which is due to the ordering of the films as well as the desorption of excess $\hbox {Bi}_2\hbox {Te}_3$ fragments from the film surface.

We performed ARPES and conductivity experiments in different ultrahigh vacuum (UHV) chambers with base pressure less than $1\times 10^{-8}$ Pa. All ARPES measurements were performed at room temperature by a R3000 electron energy analyzer (VG Scienta) and monochromatized He I$\alpha$ radiation ($h\nu =21.2\,\hbox {eV}$). In situ electrical conductivity measurements were carried out by using a home-built 4PP with a probe spacing of 0.8 mm. The 4PP was put almost in the center of a sample with the size of $10\times 4\,\hbox {mm}^2$. A Si(111) substrate was replaced by a new one after each 4PP measurement because the contact marks of the 4PP affected the uniformity of the film formed subsequently. Resistance was obtained from the slope of *I*–*V* curves measured in $|I|<1\, \upmu \hbox {A}$, and converted into sheet resistivity $\rho$ by multiplying by a geometrical correction factor^33^. All-electron band calculation was done for free-standing $\hbox {Bi}_2\hbox {Te}_3$ films by the WIEN2k code^34^ implemented with the full-potential linearized augmented plane wave plus local orbitals (APW+lo) method and the PBE96 generalized gradient approximation (GGA)^35^.

## Data Availability

The authors declare that the data supporting the findings of this study are available within the paper.

## References

[1] Geim AK, Van der Grigorieva IV (2013). Waals heterostructures. Nature.

[2] Novoselov K, Mishchenko A, Carvalho A, Neto AC (2016). 2D materials and van der Waals heterostructures. Science.

[3] Guo Y, Liu Z, Peng H (2015). A roadmap for controlled production of topological insulator nanostructures and thin films. Small.

[4] Mak KF, Lee C, Hone J, Shan J, Heinz TF (2010). Atomically thin $$\text{MoS}_2$$: a new direct-gap semiconductor. Phys. Rev. Lett..

[5] Splendiani A (2010). Emerging photoluminescence in monolayer $$\text{ MoS}_2$$. Nano Lett..

[6] Nakajima S (1963). The crystal structure of $$\text{ Bi}_2\text{ Te}_{3-x}\text{ Se}_x$$. J. Phys. Chem. Solids.

[7] Atkins P, Overton T (2010). Shriver and Atkins’ inorganic chemistry.

[8] Goldsmid HJ (2014). Bismuth telluride and its alloys as materials for thermoelectric generation. Materials.

[9] Chen Y (2009). Experimental realization of a three-dimensional topological insulator, $$\text{ Bi}_2\text{ Te}_3$$. Science.

[10] Zhang H (2009). Topological insulators in Bi$$_2$$Se$$_3$$, $$\text{ Bi}_2\text{ Te}_3$$ and $$\text{ Sb}_2\text{ Te}_3$$ with a single Dirac cone on the surface. Nat. Phys..

[11] Michiardi M (2014). Bulk band structure of $$\text{ Bi}_2\text{ Te}_3$$. Phys. Rev. B.

[12] Satterthwaite C, Ure R (1957). Electrical and thermal properties of $$\text{ Bi}_2\text{ Te}_3$$. Phys. Rev..

[13] Miller G, Li C-Y (1965). Evidence for the existence of antistructure defects in bismuth telluride by density measurements. J. Phys. Chem. Solids.

[14] Cho S (1999). Antisite defects of $$\text{ Bi}_2\text{ Te}_3$$ thin films. Appl. Phys. Lett..

[15] Hashibon A, Elsässer C (2011). First-principles density functional theory study of native point defects in $$\text{ Bi}_2\text{ Te}_3$$. Phys. Rev. B.

[16] Scanlon D (2012). Controlling bulk conductivity in topological insulators: key role of anti-site defects. Adv. Mater..

[17] Chuang P-Y (2018). Anti-site defect effect on the electronic structure of a $$\text{ Bi}_2\text{ Te}_3$$ topological insulator. RSC Adv..

[18] Li Y-Y (2010). Intrinsic topological insulator $$\text{ Bi}_2\text{ Te}_3$$ thin films on Si and their thickness limit. Adv. Mater..

[19] Zhang J (2011). Band structure engineering in $$(\text{ Bi}_{1- x}\text{ Sb}_x)_2\text{ Te}_3$$ ternary topological insulators. Nat. Commun..

[20] Liu Y, Bian G, Miller T, Bissen M, Chiang T-C (2012). Topological limit of ultrathin quasi-free-standing $$\text{ Bi}_2\text{ Te}_3$$ films grown on Si (111). Phys. Rev. B.

[21] Hoefer K (2014). Intrinsic conduction through topological surface states of insulating $$\text{ Bi}_2\text{ Te}_3$$ epitaxial thin films. Proc. Natl. Acad. Sci..

[22] Zhou G, Wang D (2015). Few-quintuple $$\text{ Bi}_2\text{ Te}_3$$ nanofilms as potential thermoelectric materials. Sci. Rep..

[23] Ghaemi P, Mong RS, Moore JE (2010). In-plane transport and enhanced thermoelectric performance in thin films of the topological insulators $$\text{ Bi}_2\text{ Te}_3$$ and $$\text{ Bi}_2\text{ Se}_3$$. Phys. Rev. Lett..

[24] Tanikawa T, Matsuda I, Kanagawa T, Hasegawa S (2004). Surface-state electrical conductivity at a metal-insulator transition on silicon. Phys. Rev. Lett..

[25] Wells JW, Kallehauge JF, Hansen TM, Hofmann P (2006). Disentangling surface, bulk, and space-charge-layer conductivity in $$\text{ Si }(111)-(7\times 7)$$. Phys. Rev. Lett..

[26] Chen C (2013). Tunable Dirac Fermion dynamics in topological insulators. Sci. Rep..

[27] Tamtögl A (2017). Electron–phonon coupling and surface Debye temperature of $$\text{ Bi}_2\text{ Te}_3$$(111) from helium atom scattering. Phys. Rev. B.

[28] Heid R, Sklyadneva IY, Chulkov EV (2017). Electron–phonon coupling in topological surface states: the role of polar optical modes. Sci. Rep..

[29] Delves R, Bowley A, Hazelden D, Goldsmid H (1961). Anisotropy of the electrical conductivity in bismuth telluride. Proc. Phys. Soc..

[30] Yavorsky BY, Hinsche N, Mertig I, Zahn P (2011). Electronic structure and transport anisotropy of $$\text{ Bi}_2\text{ Te}_3$$ and $$\text{ Bi}_2\text{ Te}_3$$. Phys. Rev. B.

[31] Vaney J-B (2019). Magnetism-mediated thermoelectric performance of the cr-doped bismuth telluride tetradymite. Mater. Today Phys..

[32] Kuzumaki T (2010). Re-investigation of the Bi-induced Si (111)-($$3\times 3$$) surfaces by low-energy electron diffraction. Surf. Sci..

[33] Yamashita M, Agu M (1984). Geometrical correction factor for semiconductor resistivity measurements by four-point probe method. Jpn. J. Appl. Phys..

[34] Blaha P (2018). WIEN2k: an augmented plane wave plus local orbitals program for calculating crystal properties.

[35] Perdew JP, Burke K, Ernzerhof M (1996). Generalized gradient approximation made simple. Phys. Rev. Lett..

